# Metformin May Improve Intestinal Mucosal Barrier Function and Help Prevent and Reverse Colorectal Cancer in Mice

**DOI:** 10.7150/jca.101251

**Published:** 2025-08-11

**Authors:** Ruiqi Wang, Longke Xie, Ping Jiang, Yaping Hou, Dazhou Li, Wen Wang

**Affiliations:** 1 Fuzong Clinical Medical College of Fujian Medical University, Fuzhou 350025, China.; 2 Department of Gastroenterology, The 900th Hospital of PLA Joint Logistic Support Force, Fuzhou 350000, China.; 3 Department of Gastroenterology, Xiamen Humanity Hospital, Xiamen 361006, China.

**Keywords:** metformin, intestinal mucosal barrier, ZO-1, occludin, colorectal cancer

## Abstract

Metformin may help prevent the development of colorectal cancer (CRC); however, the mechanisms involved remain unclear. This study aimed to investigate the effects of metformin on CRC onset and progression in a mouse model by evaluating any changes to the intestinal mucosal barrier. Sixty BALB/C female mice were randomly divided into control, model, and low-, medium-, and high-dose treatment groups. The CRC models were induced by azoxymethane combined with dextran sulfate sodium. At the time of induction, metformin 125 mg/kg · d, 250 mg/kg · d, and 500 mg/kg · d doses were administered to the low-, medium-, and high-dose groups, respectively. After 14 weeks, no tumor was observed in the control group, and multiple tumors were observed in the four test groups. Fewer tumors emerged in the metformin groups than in the model group. The tumors in the metformin groups were smaller than those in the model group. The expression of ZO-1 and occludin in the colon tissue of mice improved after metformin intervention. We performed intervention studies with varying doses of metformin and a composite disease model (parallel induction of intestinal barrier damage and tumorigenesis) in our experimental design, allowing for novel insights into the temporal effects of metformin. Metformin can improve intestinal mucosal barrier function by restoring the expression of intestinal tight junction proteins in mice and thus may help protect against CRC within a certain dose range.

## 1. Introduction

Colorectal cancer (CRC) incidence and mortality rates rank third and second among malignant tumors worldwide, respectively 1. The use of aspirin and nonsteroidal anti-inflammatory drugs may help prevent CRC 2-4. Recently, metformin has been added to this list of drugs 5 after some evidence suggested that it helps prevent carcinogenesis, regulate immunity, and delay aging 6,7. Some meta-analyses have shown that metformin can reduce the risk of tumor development 8-10, significantly reducing the incidence of colorectal adenoma and CRC while improving overall and CRC-specific survival rates 11. A carefully designed cohort study reported an inverse association between chronic metformin use and CRC risk 12 in patients with diabetes. In addition, in a randomized controlled multicenter trial, the incidence of colorectal adenomas was significantly reduced in patients without diabetes taking low-dose metformin 13. Despite this increasing evidence suggesting metformin efficacy in preventing tumors, the mechanisms involved remain unclear.

The disruption of the intestinal mucosal barrier is a major feature of tumor cell formation 14 and is frequently accompanied by the alteration or loss of tight junction protein function in tumor cells, particularly in cancers with high metastatic potential. For example, ZO-1 and occludin levels decrease during tumor formation and metastasis 15, 16.

In this study, we aimed to establish a mouse model of CRC and administer metformin to observe the development of tumors. Changes in the expression of intestinal tight junction proteins in tumor tissues were examined, and intestinal mucosal barrier function was evaluated to help elucidate the anti-tumor mechanisms involved. This evidence may support the development of novel approaches to CRC prevention and management.

## 2. Materials and Methods

### 2.1. Experimental animals and groups

Sixty SPF BALB/c female mice, 6 weeks old and weighing 10-15 g, were randomly divided into control, model, and low-, medium-, and high-dose metformin groups, using a complete randomized controlled method, allocating 12 mice per group.

### 2.2. Establishment of CRC mouse model

The study lasted 14 weeks. The mice were adaptively fed in the first week. Mice in the model and low-, medium-, and high-dose groups were intraperitoneally injected with 10 mg/kg azomethane oxide (AOM) on the first day of the second week. Subsequently, 2% dextran sulfate sodium (DSS) was dissolved in sterile drinking water in the fourth, seventh, and tenth weeks. For the remaining experimental time, the mice were fed with sterile drinking water. All mice were sacrificed at the end of week 14.

### 2.3. Intervention measures and observation indicators

Intragastric administration was started in each group simultaneously as AOM was injected intraperitoneally into the mice. Mice in the control and model groups were intragastrically administered 0.4 ml/mouse · day of sterile saline, and those in the low-, medium-, and high-dose intervention groups were intragastrically administered with metformin hydrochloride at doses of 125 mg/kg · d, 250 mg/kg · d, and 500 mg/kg · d, respectively (all dissolved in 0.4 ml/d sterile saline). The intervention was completed after three circulating 2% DSS cycles, and the mice were sacrificed at the end of week 14. Colon tumor specimens were collected and fixed in a 4% paraformaldehyde solution. Colon specimens were embedded and sectioned within 12-24 h for subsequent studies.

The morphology, hair state, mental status, and activity of the mice in each group were observed before and after the experiment. The body weights of the mice were measured and recorded weekly during the experiment. At the end of the experiment, fasting blood glucose, serum insulin, and insulin resistance levels were measured; the size and number of colorectal tumors in the mice were recorded; and the expression levels of ZO-1 and occludin proteins were detected using hematoxylin and eosin and immunohistochemical staining.

### 2.4. Statistical analysis

Data were expressed as means and standard deviation (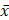
±s), and all statistical analyses were performed using IBM SPSS Statistics for Windows, version 22.0 (IBM Corp., Armonk, N.Y., USA). Continuous variables with homogeneous variance were compared among the groups using the one-way analysis of variance. The least significant difference test was used for multiple comparisons among the groups. The Kruskal-Wallis test was used to compare the variables that did not meet the homogeneity of variance assumption. Statistical significance was set at P< 0.05.

## 3. Results

### 3.1. Mouse general condition

**Figure 1:**
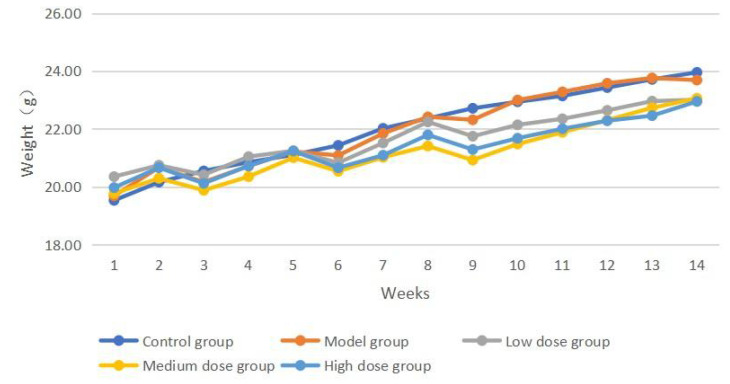
Weekly weight change curves of mice in each group,

The baseline body weight was comparable across the five groups; however, after 14 weeks of intervention, the mice in the control group showed normal growth and development, normal feeding and behavioral patterns, normal defecation, and no hematochezia. The mice in the model, low-, medium-, and high-dose treatment groups did not die during the modeling period. However, the mice in the four groups showed weight loss after each DSS administration, and some mice in all four groups showed hematochezia after the second and third DSS doses. Over time, the mouse body weight increased (Figure 1). At the end of the experiment, the body weights in all groups were comparable (Table 1).

### 3.2. Number and size of intestinal tumors in mice

**Figure 2:**
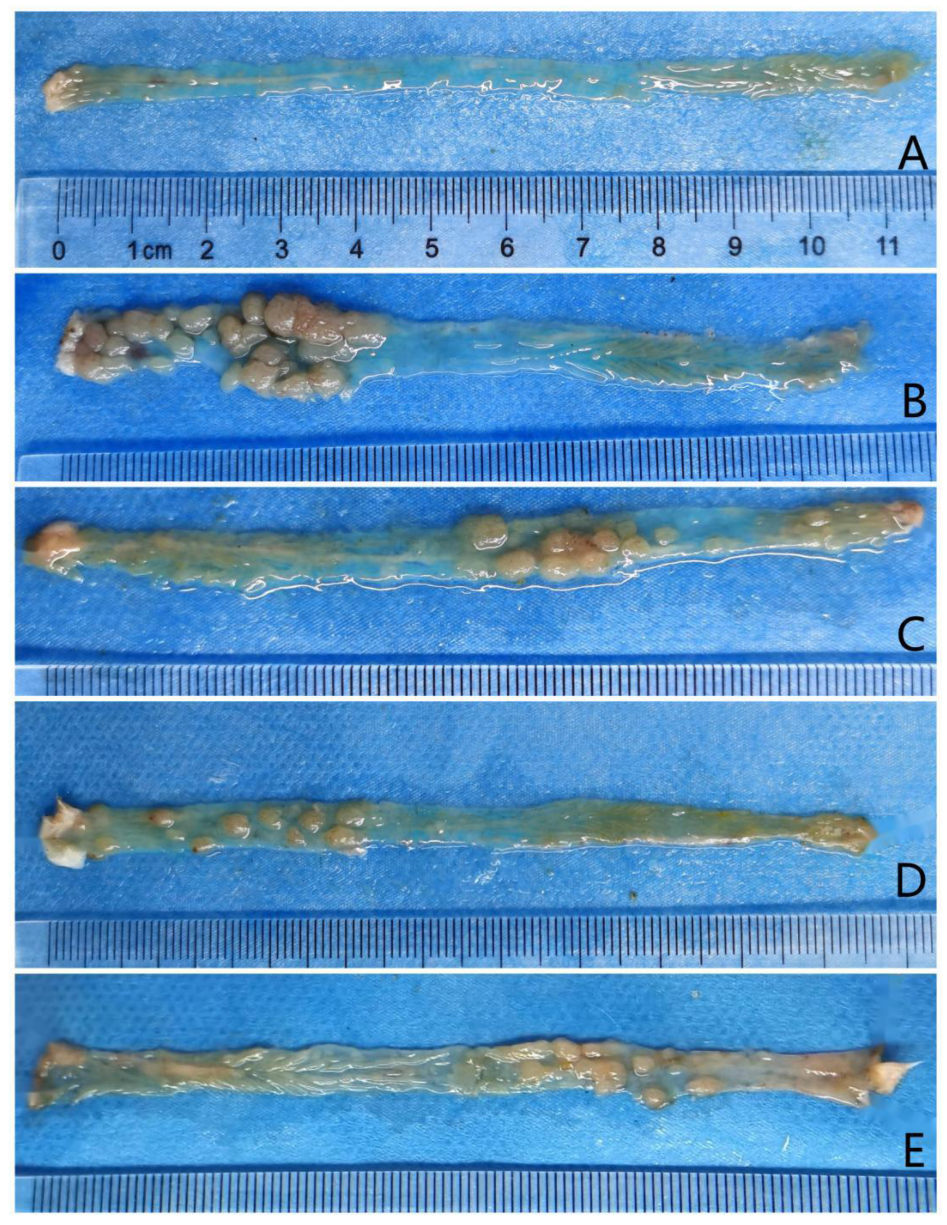
Tumors observed after longitudinal colorectal incisions in each group. Panels A, B, C, D, and E represent the control, model, and low-, medium-, and high-dose metformin groups, respectively.

Mice in the model and low-, medium-, and high-dose intervention groups were sacrificed after 14 weeks of treatment. Tumor formation was observed in the colons of mice in all four groups (Figure 2). All 12 mice in the model group had tumor formation, with an average number of intestinal tumors of 11.58 ± 7.13 and an average tumor diameter of 2.88 ± 0.35 mm. Eleven mice in the low-dose group had colon tumors, with an average number of intestinal tumors of 7.82 ± 3.54 and an average tumor diameter of 2.38 ± 0.35 mm. Ten mice in the medium-dose group had intestinal tumors, with an average number of intestinal tumors of 3.20 ± 2.25 and an average tumor diameter of 2.28 ± 0.51 mm. Twelve mice in the high-dose group had tumor formation, with an average number of intestinal tumors of 3.58 ± 2.35 and an average tumor diameter of 2.23 ± 0.42 mm (Table 2).

**Figure 3:**
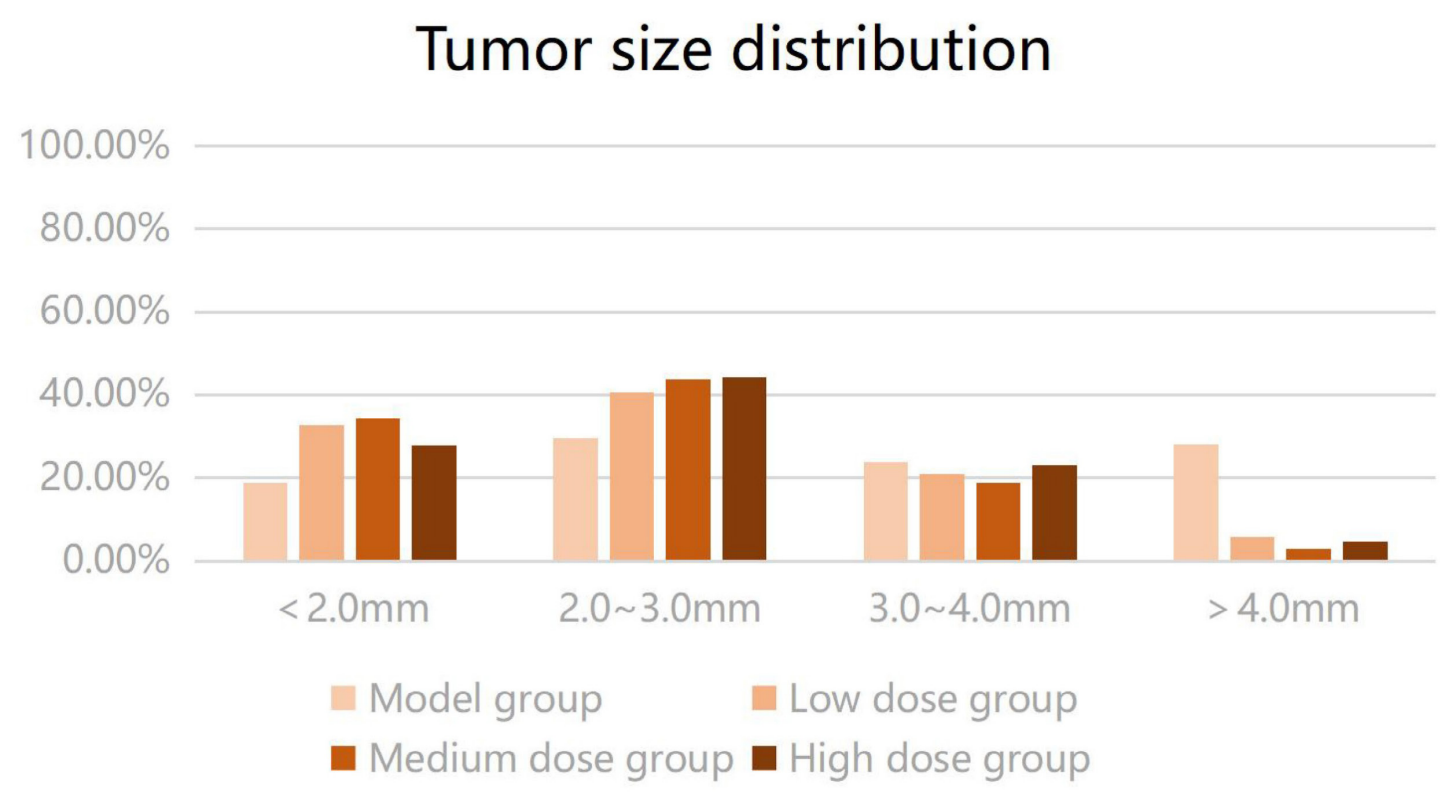
Tumor diameter (mm) distribution in each group.

The number of intestinal tumors in the three groups of mice after metformin intervention was lower than that in the model group (P < 0.05). The number of tumors in the low-dose group was higher than that in the medium- and high-dose groups, and the number of tumors in the medium- and high-dose groups was lower than that in the low-dose group (P < 0.05); meanwhile, the number of tumors was comparable in the medium- and high-dose groups (P > 0.05). Metformin reduced the number of colon tumors in mice, and the effect was greater when the dose was increased within a certain range. The average intestinal tumor diameter of the mice receiving metformin was smaller than that of the model group; however, no significant difference was found in the mean diameter of intestinal tumors among the three metformin groups (P > 0.05). The results suggested that metformin could reduce the size of colon tumors in mice; however, no dose-response relationship was observed. The size distribution of intestinal tumors is shown in Figure 3. The tumor diameters of mice in the low-, medium-, and high-dose intervention groups were smaller than those in the model group; particularly, the proportion of tumors with a diameter of > 3 mm was significantly reduced compared with that in the model group. Therefore, we compared the proportion of tumors with a diameter of > 3 mm in the four groups of mice using the Kruskal-Wallis H test. Compared to the model group, a statistically significant difference was found in the proportion of tumors with a diameter of > 3 mm in each metformin group (H = 12.517, P < 0.05). Metformin reduced the number of colorectal tumors to > 3 mm in diameter in CRC mice.

### 3.3. Blood glucose and insulin resistance index of mice after intervention

**Figure 4:**
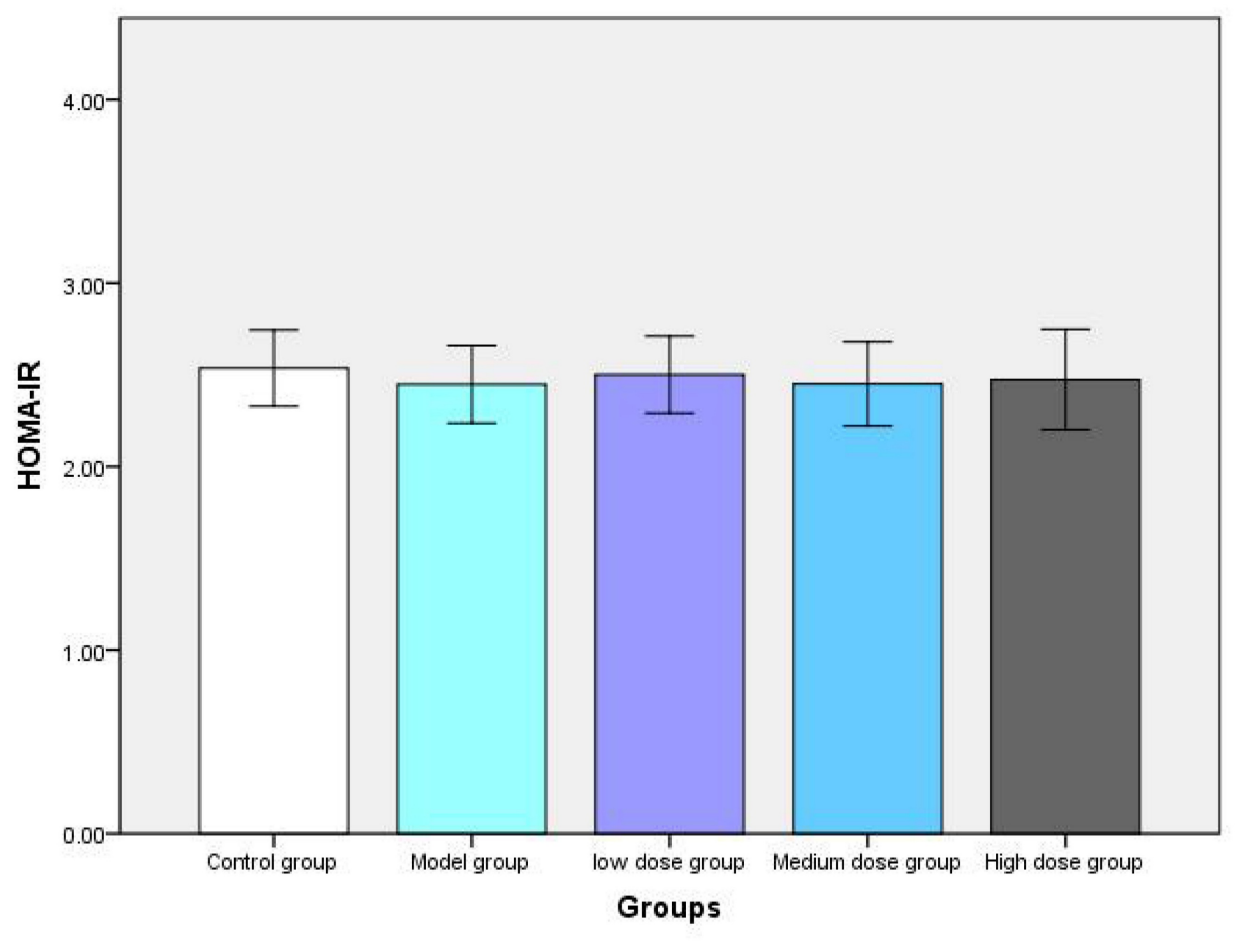
Post-experiment fasting blood glucose, serum insulin, and HOMA-IR values of mice in each group (A, B, and C represent fasting blood glucose, serum insulin, and HOMA-IR of mice, respectively).

After 14 weeks of the intervention, the insulin resistance index was comparable among the five groups (Figure 4). These results suggest that metformin does not induce hypoglycemic responses in non-diabetic mice or alter serum insulin levels or insulin resistance indices in non-diabetic mice, implying that it may be safe to use in non-diabetic mice.

### 3.4. Hematoxylin and eosin staining and immunohistochemical staining

**Figure 5:**
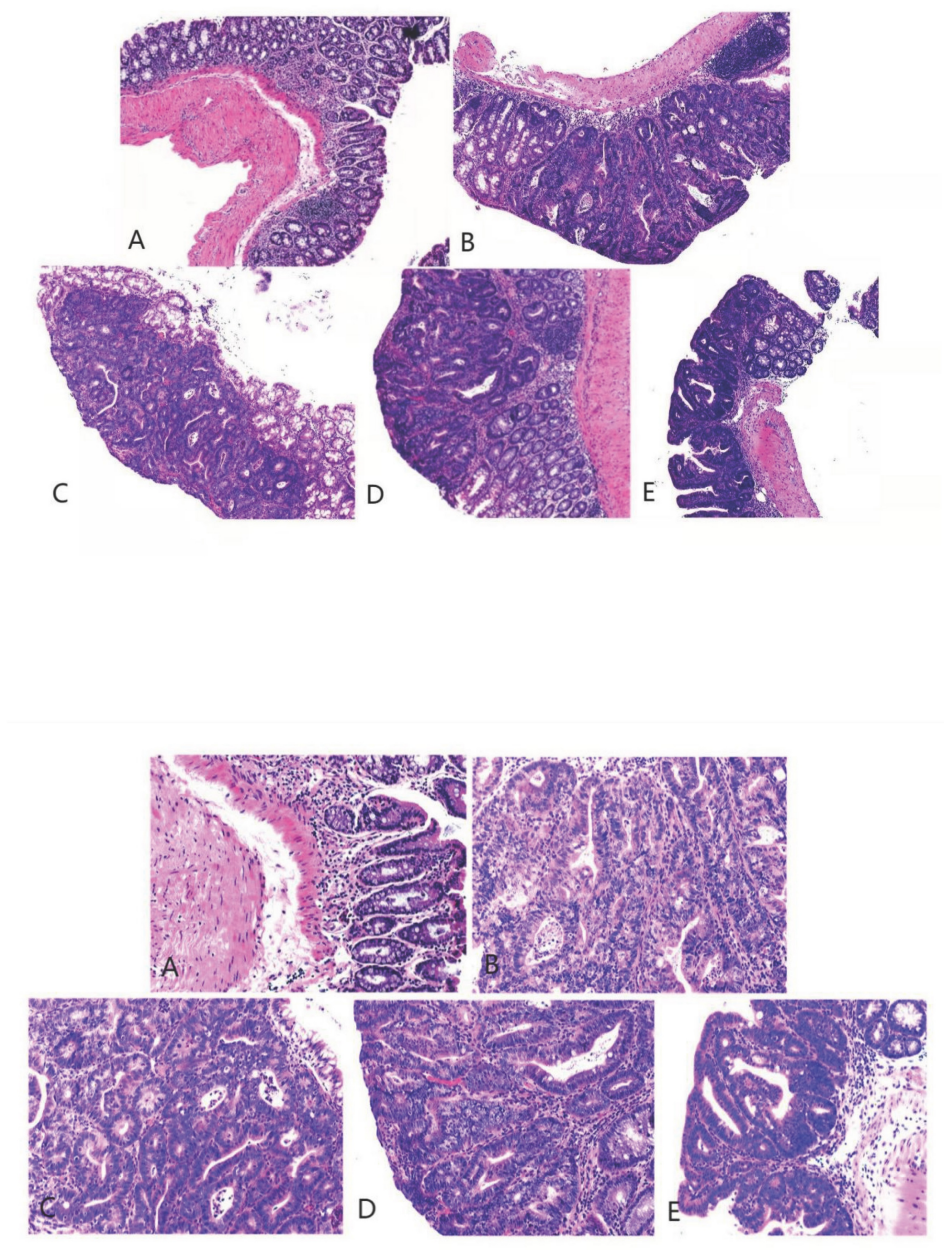
Hematoxylin and eosin staining of colon cancer tissues obtained from the mice (magnification 40 as shown in [up] and 100 times [down]). A, B, C, D, and E represent the control and model, and low-, medium-, and high-dose groups treated with metformin, respectively.

**Figure 6:**
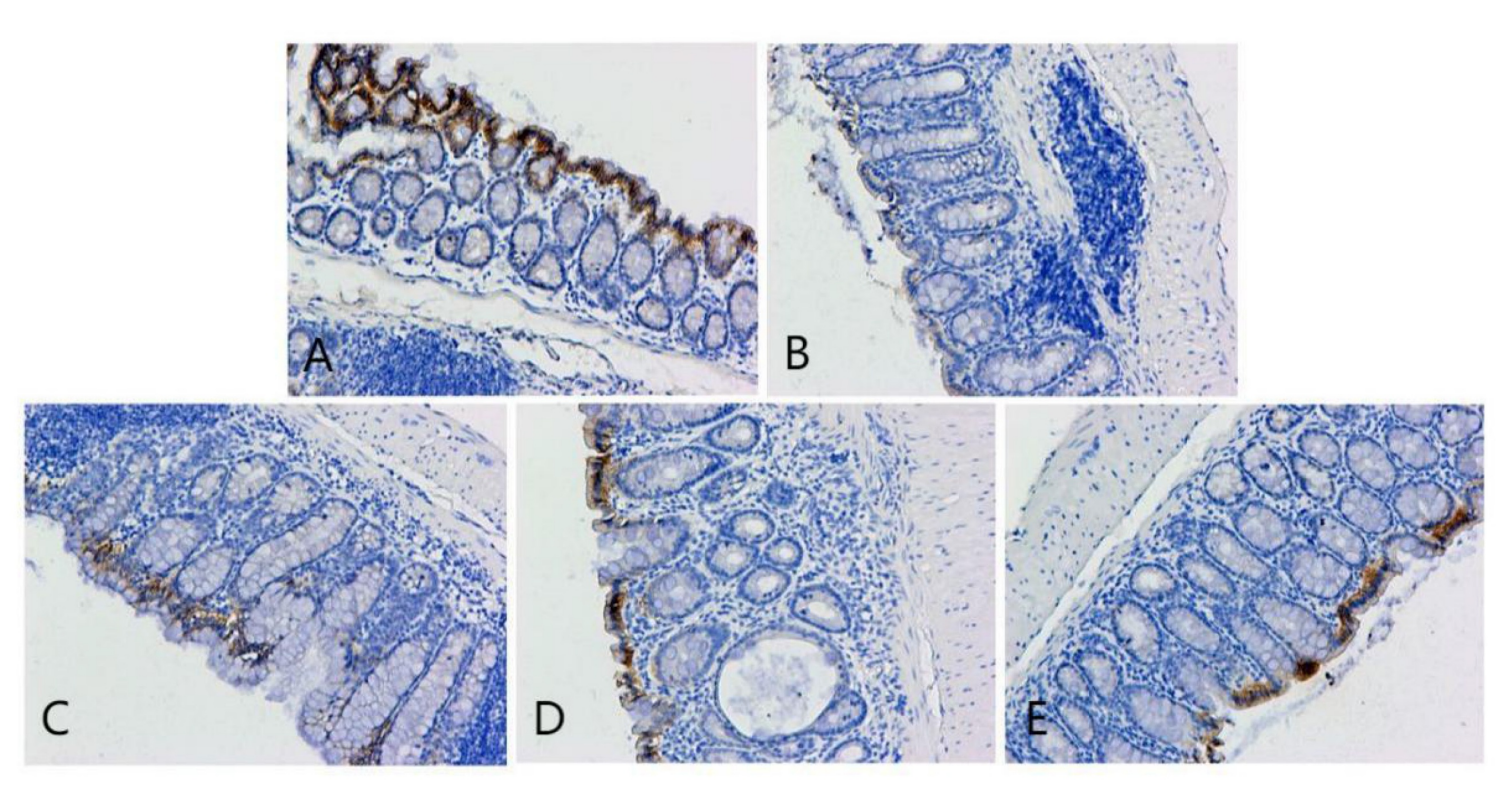
OCLN immunohistochemical staining of colonic lesion tissue samples of mice in each group. A, B, C, D, and E represent the control and model, and low-, medium-, and high-dose groups treated with metformin, respectively.

**Figure 7:**
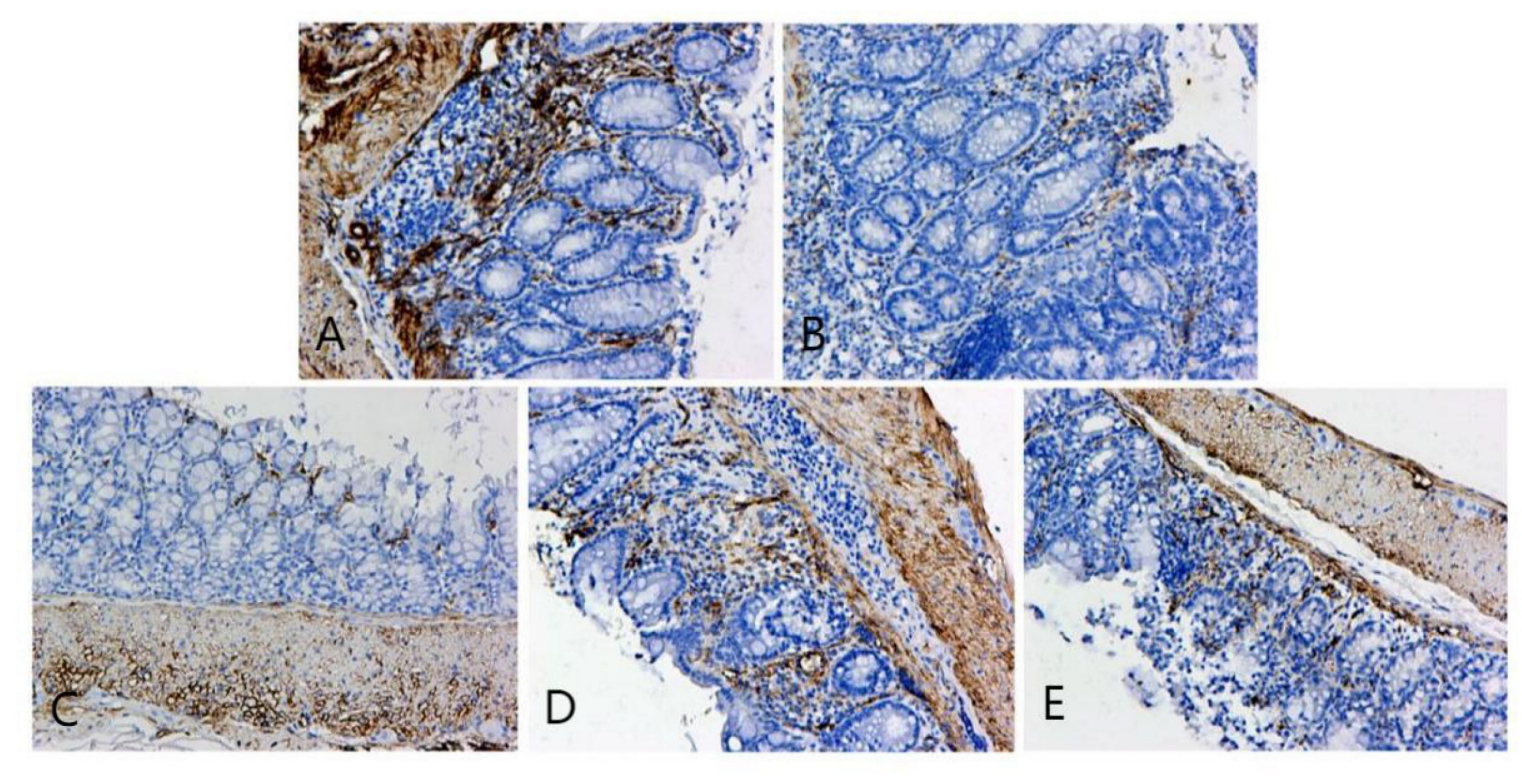
ZO-1 immunohistochemical staining of colon cancer tissues of mice in each group. A, B, C, D, and E represent the control and model, and low-, medium-, and high-dose groups treated with metformin, respectively.

**Figure 8:**
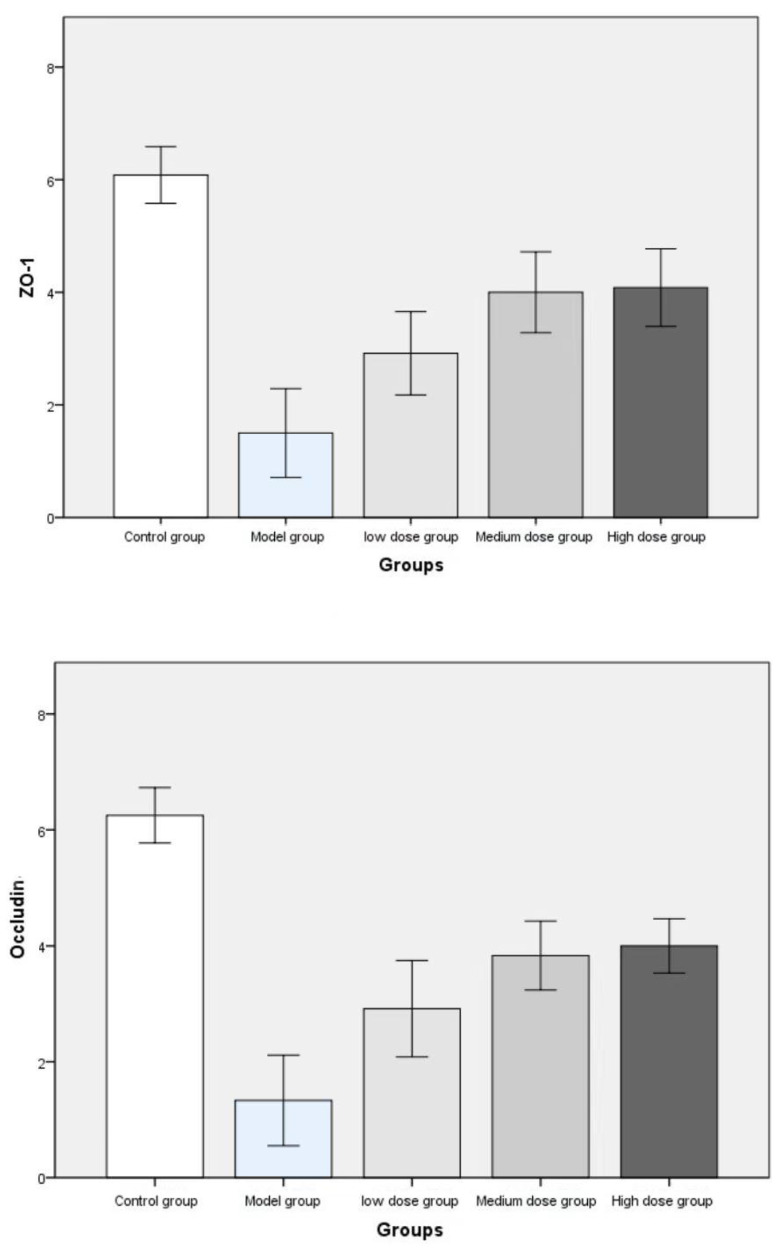
Immunohistochemical staining scores of ZO-1 and occludin were compared among the groups.

The pathological findings were tubular adenoma with low-grade intraepithelial neoplasia and carcinogenesis (Figure 5). The expression levels of ZO-1 and occludin in the colon tissues of mice in the four experimental groups decreased. In the model group, the expression of occludin and ZO-1 proteins significantly decreased. The expression levels of ZO-1 increased with the increase in the dose of metformin. However, no significant statistical difference was found between the medium- and high-dose groups. The results showed that the intestinal mucosal barrier of the CRC model mice was damaged to a different extent in each group. The damage in the model control group was the most extensive, followed by that in the low-, medium- and high-dose groups; however, these differences were not statistically significant (Figures 6 to 8).

## 4. Discussion

Metformin may have pleiotropic effects beyond glycemic control by activating the AMPK pathway 17, 18. However, the molecular mechanism by which metformin affects blood glucose levels remains unclear. The first molecular target for metformin was proposed in a 2022 study that indicated PEN2-ATP6AP1 as a mediator of metformin in the AMPK pathway 19. Follow-up studies have shown that low-dose metformin acting on the AMPK-PEN2-ATP6AP1 axis can reduce postprandial blood glucose and liver fat levels and prolong lifespan. However, it remains unclear whether this target is associated with any anti-tumor effects. Some evidence suggests that metformin may have anti-cancer properties 20-22, although the mechanisms involved remain unclear. Meanwhile, some investigators believe that hyperinsulinemia promotes tumorigenesis by acting on insulin receptors in the epithelium or by affecting insulin-like growth factor pathways, inflammation, or adipokine-induced cancer cell proliferation and metastasis 23, while metformin may provide a preventive effect by reducing insulin resistance and hyperinsulinemia 24. Another important pathway related to cancer growth is the mammalian target of the rapamycin (mTOR) pathway 25, which promotes cell growth and division and supports angiogenesis and benign-to-malignant cell transformation. The central role of the mTOR pathway in cell growth and division makes it a potential target for anti-tumor drugs. Metformin may help prevent and reduce tumor growth by inhibiting the mTOR pathway 20. It has been shown 26 that low or intermittent administration of mTOR inhibitors has an anti-tumor effect, while excessive doses of mTOR inhibitors exert a strong immunosuppressive effect. In this study, metformin did not demonstrate dose-response anti-tumor effects beyond a certain range. In addition, metformin combined with some chemotherapies can show synergistic anti-cancer effects while reducing treatment side effects 27.

However, the role of metformin in CRC carcinogenesis in patients without diabetes remains unclear. CRC is associated with mucosal barrier dysfunction. Physiological mucosal barrier function has been linked to tight junctions 16, whose abnormal expression can induce changes in intestinal permeability and contribute to tumor development and invasion of colonic epithelial cells 28, 29. Herein, we demonstrated that metformin can improve intestinal mucosal barrier function by affecting the expression of tight junction proteins in the intestinal mucosa, including occludin, ZO-1, and claudins, which are closely related to the development of CRC. Occludin and ZO-1 protein expression in the intestinal mucosal barrier is suppressed during the onset and development of some malignancies and is related to the progression of malignant tumors 30. The CRC mouse model induced by AOM/DSS simulated the CRC formation process observed in humans. In this study, all experimental groups developed colon tumors, whose size and number were reduced after treatment with metformin, which helped recover the mucosal barrier function. Some dose-response effects were observed in the range of 125-250 mg/day; however, higher doses were not any more effective at reducing tumor size or number or at improving the mucosal barrier function. This evidence suggests that the mucosal barrier is disrupted in CRC and that the use of metformin in non-diabetic mice is relatively safe when considering glycemic control. Overall, this evidence suggests that the use of metformin in non-diabetic mice could help prevent the onset and progression of CRC by improving the function of the intestinal mucosal barrier. However, further studies are required to validate these findings and examine whether tight junction expression may be a tumor marker in CRC.

The above results show that metformin can prevent CRC by improving the intestinal mucosal barrier function; however, this experiment also has the following limitations: first, insufficient sample size, and the possible experimental error; second, the selection of metformin dose was not detailed, and the starting and optimal doses of metformin had not been explored; third, the molecular mechanism of metformin improving metformin, requiring further exploration; fourth, the molecular mechanism for the occurrence and development of tight junctions and CRC remains to be further clarified. It is believed that with the development of molecular biology technology, the understanding of tight junctions and the development of intestinal tumors will be further deepened; the anti-tumor mechanism of metformin will be further explored, which will be more meaningful for preventing and treating clinically relevant diseases.

## 5. Conclusions

Non-diabetic mice treated with metformin showed reduced number and diameter of CRC tumors induced by AOM/DSS compared to those in the control group. The anti-tumor effects of metformin may follow a dose-response relationship within a certain value range, and their efficacy may be related to improved tight junctions in the intestinal mucosal barrier. Further studies are required to validate these results.

## Figures and Tables

**Table 1:** Body weight of each group before and after intervention (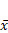
±s)

Group	Number of mice	Pre-intervention weight (g)	Post-intervention weight (g)
Control group	12	19.53±1.09	23.96±1.02
Model group	12	19.69±0.96	23.69±1.00
Low dose group	12	20.35±0.82	23.01±0.96
Medium dose group	12	19.07±1.07	23.06±1.18
High dose group	12	19.97±1.22	22.95±1.42

**Table 2:** Average number and diameter of intestinal tumors observed in each group

Groups	Number of CRC mice	Mean number of tumors	Mean tumor diameter (mm)
Control group	0	0	0
Model group	12	11.58±7.13	2.88±0.35
Low dose group	11	7.82±3.54	2.38±0.35
Medium dose group	10	3.20±2.25	2.28±0.51
High dose group	12	3.58±2.35	2.23±0.42

CRC, colorectal cancer

## Abbreviations

CRC: colorectal cancer
AOM: azomethane oxide
DSS: dextran sulfate sodium
